# Effects of NaCl concentration on anode microbes in microbial fuel cells

**DOI:** 10.1186/s13568-015-0123-6

**Published:** 2015-06-11

**Authors:** Morio Miyahara, Atsushi Kouzuma, Kazuya Watanabe

**Affiliations:** School of Life Sciences, Tokyo University of Pharmacy and Life Sciences, 1432-1 Horinouchi, Hachioji, Tokyo 192-0392 Japan

**Keywords:** Microbial electrochemical cell, Ionic strength, Real-time PCR, 16S rRNA gene, Phylogenetic analysis, Exoelectrogen

## Abstract

Understanding of how operational parameters affect the composition of exoelectrogenic microbes is an important step in the development of efficient microbial fuel cells (MFCs). In the present study, single-chamber MFCs were inoculated with rice paddy-field soil and continuously supplied with an acetate medium containing different concentrations of NaCl (0–1.8 M). Polarization analyses showed that power output increased as the NaCl concentration increased to 0.1 M, while it was markedly diminished over 0.3 M. The increase in power output was associated with an increased abundance of anode microbes as assessed by protein assays. Notably, the power increase was also accompanied by an increase in the abundance ratio of *Geobacter* bacteria to total anode bacteria as assessed by pyrosequencing of 16S rRNA gene amplicons and specific quantitative PCR. Although most *Geobacter* species are known to exhibit high growth rates in freshwater media without NaCl, the present study shows that 0.1 M NaCl facilitates the growth of *Geobacter* in MFC anode biofilms. This result suggests that the optimum salt concentration in MFC is determined by the balance of two factors, namely, the solution conductivity and salt tolerance of exoelectrogens.

## Introduction

Microbial fuel cells (MFCs) are devices that use living microbes for the generation of electricity coupled to the decomposition of organic matter (Logan et al. 2006; Watanabe 2008; Pant et al. 2010). Owing to the great diversity of microbial metabolic capacities, MFCs are capable of generating electricity from a wide range of organic and inorganic compounds. Furthermore, MFCs can generate electricity from biomass waste and pollutants in wastewater by exploiting naturally occurring microbial communities as self-organizing anode catalysts (Rozendal et al. 2008; Lefebvre et al. 2011). Due to these advantageous properties, extensive efforts are being made to develop MFCs as energy-saving and cost-efficient options for wastewater treatment (Du et al. 2007; Lefebvre et al. 2011).

For MFCs to be practically applied to renewable energy generation, several factors need to be improved, particularly power outputs. Power outputs from MFCs are affected by numerous factors, including cell configuration, electrode materials, microbial inocula, substrates, and electrolyte compositions (Kim et al. 2007; Rinaldi et al. 2008). Among these factors, the composition of electrolyte has been shown to critically affect various aspects of MFC performance. For instance, proton carriers, such as phosphate and carbonate ions, improve the kinetics of proton transfer, resulting in enhanced power output (Fan et al. 2007). Electrolyte salt concentrations (correlated with ionic strength) have also been shown to affect MFC power output (Liu et al. 2005; Heilmann and Logan 2006; Mohan and Das 2009; Lefebvre et al. 2012; Rousseau et al. 2013). The findings from these studies are useful for the development of MFCs for wastewater treatment, as wastewater salinity varies markedly depending on the geographical region (Lefebvre and Moletta 2006; Lefebvre et al. 2012). In addition, the salt concentration of the aqueous phase may influence microbial metabolic activities (McCarty and McKinney 1961). Although the potential effects of salt concentration on microbes, including exoelectrogens, in MFCs have previously been discussed (Lefebvre et al. 2012), no studies have examined the effects of salt concentration on anode microbes in MFCs.

The present study was undertaken to examine the effects of different concentrations of NaCl on anode microbes and power generation of MFCs. MFCs were inoculated with rice paddy-field soil and operated at NaCl concentrations ranging from 0 to 1.8 M for examining potential interdependencies among NaCl concentration, power output, and anode microbes.

## Materials and methods

### Reactor configuration and operation

**Figure 1:**
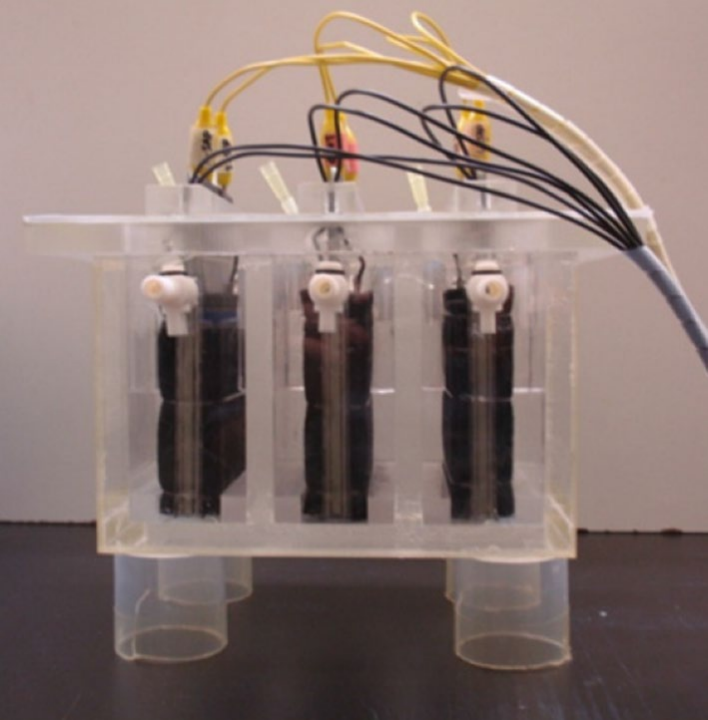
Photograph of the MFC box composed of three independent cassette-electrode MFCs used in this study. Each MFC unit consisted of a cassette electrode, and had a water inlet and outlet positioned on opposite sides of the unit.

The MFCs used in the present study are shown in Figure 1. Three MFC units were housed in a single MFC box but were operated independently. Each unit was equipped with a cassette electrode (Shimoyama et al. 2008) which consisted of two sets of air cathodes (Cheng et al. 2006), separators and graphite felt anodes (5-mm thickness; Sohgoh Carbon, Yokohama, Japan) and were prepared as described previously (Miyahara et al. 2013). The anode and cathode had projected areas of 68 and 65 cm^2^, respectively. The liquid capacity of each unit was approximately 300 mL. The liquid surface was covered with a polystyrene board, and the reactor boxes were placed in a water bath at 30°C during operation. MFCs were continuously supplied with an acetate medium (pH 7.0) containing (per liter) 820 mg sodium acetate (10 mM), 50 mg BBL yeast extract, 175 mg NH_4_Cl, 5.26 mg KH_2_PO_4_, 22.05 mg CaCl_2_·2H_2_O, 0.43 mg MgSO_4_·7H_2_O, 21.3 mg KCl, 8.76 mg NaHCO_3_, and 1 mL of trace element solution (DSMZ 663; Deutsche Sammlung von Mikroorganismen und Zellkulturen GmbH).

The operation of MFCs was initiated by inoculating each reactor with 1 g rice paddy-field soil (collected from Noda, Chiba, Japan) and supplying the acetate medium at a flow rate of 300 ml day^−1^, corresponding to a hydraulic retention time of 1 day, using peristaltic pumps (SJ1220, Atto, Tokyo, Japan). The anodes and cathodes of each unit were connected via an external resister (*R*_ext_ [Ω]), and the voltage across the resister (*E* [V]) was monitored using a data logger (GL820, Graphtec, Yokohama, Japan). Current (*I* [A]) was calculated from *R*_ext_ and *E* according to the Ohm’s law (*I* = *E*/*R*_ext_).

### Chemical analyses and evaluation of MFC performance

Polarization curves were measured by linear sweep voltammetry (LSV) using a potentiostat (HZ-5000, Hokuto Denko, Tokyo, Japan) at a scan rate of 0.5 mV s^−1^, and power curves were generated based on the polarization curves (Logan et al. 2006). In these analyses, current and power densities (*J* [A m^−2^] and *P* [W m^−2^], respectively) were calculated based on the projected anode area. Open-circuit voltage (*E*_op_ [V]), maximum power density (*P*_max_), and internal resistance (*R*_int_ [Ω]) were then determined from the polarization and power curves. Acetate was measured using a high performance liquid chromatograph (1100 series; Agilent Technologies, Tokyo, Japan) equipped with a Zorbax SB-Aq column (Agilent Technologies) as described elsewhere (Newton et al. 2009).

### Analyses of anode microbiomes

Pieces of graphite felt were cut from anodes on both sides of the cassette electrode on day 82 of MFC operation and were stored at −20°C. To determine the total protein content of anode to estimate the total microbial biomass, proteins were extracted from the anode pieces (0.5 cm^2^) using B-PERII reagent (Pierce, Rockford, IL, USA) and were quantified using a BCA protein kit (Pierce) as described previously (Shimoyama et al. 2009).

DNA was extracted from the pieces of graphite-felt anodes (0.5 cm × 0.5 cm) using a Fast DNA Spin Kit for Soil (Q-Bio, Carlsbad, CA, USA) according to the manufacturer’s instruction and was finally dissolved in 50 μl of the DES solution supplied in the kit. For sequence analyses, PCR amplification of 16S rRNA gene fragments (V1–V3 region) was performed using primers ad-tag-8F (5′-CGTATCGCCTCCCTCGCGCCATCAGXXXXXXGAGTTTGATCMTGGCTCAG-3′) and ad-533R (5′-CTATGCGCCTTGCCAGCCCGCTCAGTTACCGCKRCTGCTGRCAC) (Watanabe et al. 2004), in which the underlined sequences are adaptors added for pyrosequencing and XXXXXX represents an arbitrary tag sequence for sample identification (Dowd et al. 2008). The PCR conditions were as described elsewhere (Miyahara et al. 2013), and amplicons were purified using a QIAquick PCR Purification Kit (Qiagen K. K., Tokyo, Japan). Amplicons from different samples were mixed at the same concentration (1 ng μl^−1^ each) and then subjected to pyrosequencing using a Genome Sequencer FLX system (Roche Applied Science, Tokyo, Japan). Phylogenetic analyses were conducted using the Silva rRNA database (http://www.arb-silva.de/), and a tree was constructed by the neighbor-joining method using MEGA5 (Tamura et al. 2011). Nucleotide sequences determined in the present study were deposited into the DDBJ Sequence Read Archive Database (accession numbers: DRX025202 to DRX025213 and DRR027607 to DRR027618).

The abundance ratio of *Geobacteraceae* bacteria to total bacteria in the anode microbes was evaluated by quantitative real-time PCR (qPCR), as described previously (Kato et al. 2010). Briefly, real-time PCR was performed using a LightCycler system and LightCycler DNA Master SYBR Green I kit (Roche Applied Science) according to the manufacturer’s instructions. 16S rRNA genes of *Geobacteraceae* bacteria were amplified using the primer pair Geo494F and Geo825R (Holmes et al. 2002), while those of total bacteria were amplified using the primer pair 341f and 534r (Watanabe et al. 2001). Standard curves for *Geobacteraceae* and total bacteria were generated using serially diluted genomic DNA extracted from *Geobacter sulfurreducens* (10 pg µl^−1^ to 100 ng µl^−1^). The abundance ratio was calculated by dividing the 16S rRNA gene copy number of *Geobacteraceae* bacteria by that for total bacteria.

## Results

### Effects of NaCl concentration on power output

We examined the power outputs from MFCs supplied with the acetate medium containing NaCl at concentrations of 0, 0.05, 0.1, 0.3, 0.6 and 1.8 M (0M-MFC, 0.05M-MFC, 0.1M-MFC, 0.3M-MFC, 0.6M-MFC and 1.8M-MFC, respectively). These NaCl concentrations were selected to mimic freshwater (0 M), brackish water (0.05–0.3 M), seawater (0.6 M) and hyper-saline lakes (1.8 M). Since concentrations of other electrolyte ions were low (sodium acetate [10 mM] was the highest), NaCl was the major determinant of ionic strength in the electrolyte.

**Figure 2:**
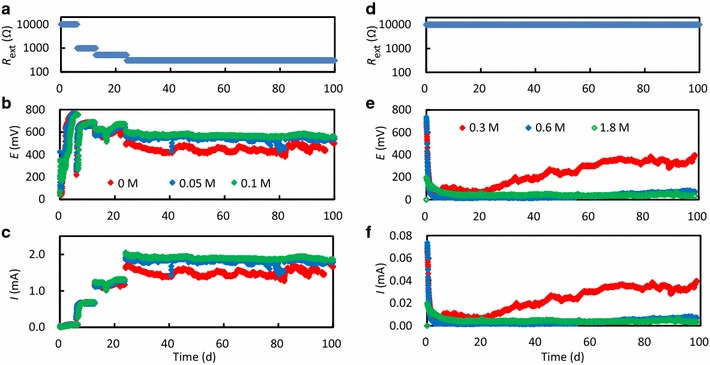
Changes in *R*_ext_ (**a**, **d**), *E* (**b**, **e**) and *I* (**c**, **f**) during the 100-day operation of the MFCs containing different NaCl concentrations. Data for the 0M-, 0.05M- and 0.1M-MFCs (*red*, *blue* and *green symbols*, respectively) are presented in **a**, **b** and **c**, while those for the 0.3M-, 0.6M- and 1.8M-MFCs (*red*, *blue* and *green symbols*, respectively) are presented in **d**, **e** and **f**.

The operation of MFCs was initiated with *R*_ext_ of 10,000 Ω, and it was decreased when *E* exceeded 600 mV (Figure 2a, d). In 0M-, 0.05M- and 0.1M-MFCs, *E* relatively rapidly increased (Figure 2b) and *R*_ext_ was finally maintained at 300 Ω (Figure 2a). In contrast, *E* slowly increased in 0.3M-MFC (Figure 2e), but *I* only reached 0.04 mA during the 100-day operation (Figure 2f). Furthermore, *E* only slightly increased in 0.6M- and 1.8M-MFCs and did not exceed 100 mV (Figure 2e). In all of the MFCs, the acetate concentration in the reactor effluent was between 1 and 5 mM; it is noteworthy that the removal of organics was partially attributable to oxygen respiration in air–cathode MFCs (Shimoyama et al. 2008). Taken together, these results indicate that the NaCl concentration largely influenced the MFC performance and should be below 0.1 M for electricity generation in MFCs inoculated with paddy-field soil.

**Figure 3:**
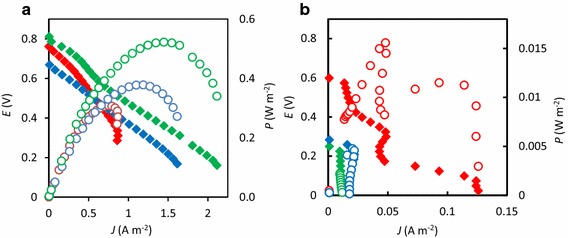
Electrochemical characterizations of the MFCs. Polarization and power curves for the 0M-, 0.05M- and 0.1M-MFCs (*red*, *blue* and *green symbols*, respectively) are shown in **a**, while those for the 0.3M-, 0.6M- and 1.8M-MFCs (*red*, *blue* and *green symbols*, respectively) are shown in **b**.

Polarization analyses were conducted once electric output of these MFCs became stable (after day 60), and representative data are presented in Figure 3. Mean polarization parameters estimated for these MFCs during day 60–100 are summarized in Table 1. Although typical polarization and power curves were obtained for the 0M-, 0.05M- and 0.1M-MFCs (Figure 3a), those for the other MFCs operated at higher NaCl concentrations were atypical (Figure 3b). In addition, *E*_op_ values of 0.3M-, 0.6M- and 1.8M-MFCs were low, suggesting that these MFCs operated poorly as fuel cells. The polarization data (Table 1) show that the MFC performance of 0M-, 0.05M to 0.1M-MFC increased with increasing NaCl concentration.

**Table 1:** Polarization parameters after electric outputs became stable

NaCl (M)	*E*_op_ (mV)	*P*_max_ (mW m^−2^)	*R*_int_ (Ω)
0	766 ± 29	114 ± 4	192 ± 15
0.05	812 ± 22	340 ± 21	83 ± 14
0.1	816 ± 18	504 ± 41	43 ± 5
0.3	555 ± 50	15.5 ± 1.4	1,102 ± 120
0.6	257 ± 15	2.4 ± 0.3	3,318 ± 200
1.8	247 ± 47	1.6 ± 0.3	1,866 ± 320

Data are mean ± SD (n = 5).

### Effects of NaCl on anode microbes

**Figure 4:**
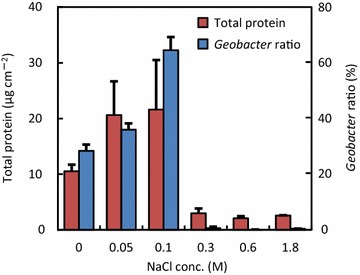
Total protein contents and abundance ratios of *Geobacteraceae* bacteria to total bacteria, as determined by qPCR for microbial samples collected from the MFC anodes.

To examine the effects of NaCl concentration on anode microbes in MFCs, we first analyzed the total protein content of anode samples as a surrogate measure of the abundance of microbes attached to the anodes. We found that microbes were the most abundant on the anodes of the 0.05M- and 0.1M-MFCs, followed by the 0M-MFC (Figure 4), whereas anode microbes in the 0.3M-, 0.6M- and 1.8M MFCs were only one tenth to one-fifth as abundant as those in the 0.1M-MFC (Figure 4). Interestingly, these findings suggest that there is a threshold NaCl concentration between 0.1 and 0.3 M that determines the growth of anode microbes in MFCs.

Analyses of MFC anode-associated microbial communities frequently detect bacteria affiliated with the family *Geobacteraceae* which includes well-characterized exoelectrogens, such as *Geobacter* (Logan 2009). In the present study, qPCR was used to examine if *Geobacteraceae* bacteria were present among anode-associated microbes in the MFCs (Figure 4). *Geobacteraceae* bacteria were substantially detected in the 0M-, 0.05M-, and 0.1M-MFCs, and their abundance ratio relative to total bacteria increased with increasing NaCl concentration, reaching over 60% in the 0.1M-MFC. However, *Geobacteraceae* bacteria were not substantially detected in MFCs with NaCl concentrations of 0.3M or higher.

**Figure 5:**
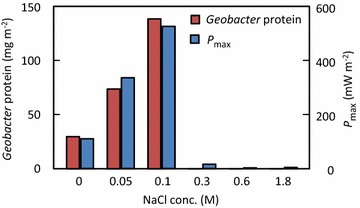
Correlation between *P*_max_ and *Geobacteraceae* protein content at different concentrations of NaCl. *Geobacteraceae* protein content was estimated by multiplying the total protein content by the abundance ratio of *Geobacteraceae* determined by qPCR.

To confirm that the observed effects of NaCl on electricity generation were associated with the growth of anode-associated *Geobacteraceae* bacteria, the abundance of *Geobacteraceae* bacteria as expressed by the *Geobacteraceae* protein content were estimated from the total-protein content and their abundance ratio, and the estimated values are compared with *P*_max_ values at the different NaCl concentrations (Figure 5). A close correlation was clearly detected between these values, confirming that *Geobacteraceae* bacteria were responsible for the MFC power generation.

**Figure 6:**
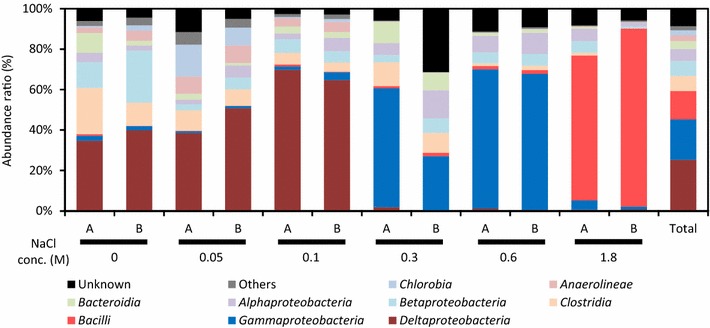
Phylogenetic distribution of bacteria based on 16S rRNA gene fragments PCR-amplified from anode biofilms in MFCs operated at different concentrations of NaCl. Abundance ratios of different groups of anode microbes classified at the class level are shown. For each sample, two patterns (*A*, *B*) are presented, which represent microbial communities in anode samples obtained from different sides of cassette electrodes.

To further characterize anode-associated microbes in the MFCs, we conducted pyrosequencing and phylogenetic analyses of PCR-amplified 16S rRNA gene fragments. Figure 6 presents the abundance ratios of bacterial groups classified at the class level in each MFC. As expected, the relative abundance of the class *Deltaproteobacteria*, which includes the family *Geobacteraceae*, increased as the NaCl concentration increased from 0 to 0.1 M; this class comprised over 60% of the total bacteria in the 0.1M-MFC. However, the community structure dramatically differed at NaCl concentrations of 0.3 M and higher; members of the class *Gammaproteobacteria* were the most abundantly detected at 0.3 and 0.6 M, whereas *Bacilli* was the most abundant at 1.8 M.

**Figure 7:**
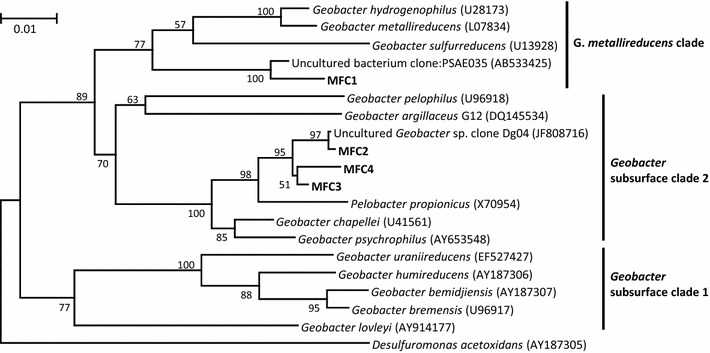
A phylogenetic tree based on partial 16S rRNA gene sequences showing taxonomic positions of the major *Geobacter* sequences (MFC1 to MFC4). Bootstrap values (100 trials, only >50 are shown) are indicated at branching points. The *bar* indicates 1% sequence divergence. Accession numbers are shown in *parentheses*.

In order to show what sequences constituted the major class-level taxonomic groups in Figure 6, major sequences (>1% to the total sequence in each library) are listed in Table 2. It is shown that the *Deltaproteobacteria* detected at 0–0.1 M NaCl are comprised of several major sequences affiliated with the genus *Geobacter*, the *Gammaproteobacteria* detected at 0.3 and 0.6 M includes major sequences affiliated with *Pseudomonas* and *Aeromomas*, while the *Bacilli* detected at 1.8 M NaCl was *Staphylococcus*. The major *Geobacter* sequences (MFC1 to MFC4) were further analyzed to identify exact phylogenetic positions (Figure 7); in this figure, *Geobacter* sequences are divided into three clades according to a previous study (Holmes et al. 2007). This analysis shows that MFC1 affiliated with the *G. metallireduces* clade was seen only at 0 M NaCl, while MFC2, the most abundant sequence at 0.05 M and 0.1 M NaCl, is affiliated with the subsurface clade 2. It is suggested that the high power density observed in 0.1M-MFC was attributed to the preferential growth of subsurface clade 2 *Geobacter* at 0.1 M NaCl.

**Table 2:** Major sequences (>1% to total) detected from anodes of MFCs at different NaCl concentrations

NaCl	Order	No. of read	Percent (%)	Closely related sequence (accession no.)	Taxonomy	Description
0 M	1	1,328	15.6	Uncultured bacterium Jan1A06 (GU139308)	*Geobacter*	MFC1
	2	1,260	14.8	*Geobacter* sp. Ply1 (EF527233)	*Geobacter*	MFC2
	3	373	4.4	Uncultured bacterium MBfR28-36 (EU169844)	*Rhodocyclaceae*	
	4	262	3.1	Uncultured bacterium IIB-27 (AJ488087)	*Rikenellaceae*	
	5	258	3.0	*Comamonas granuli* (AB187586)	*Comamonas*	
	6	249	2.9	Uncultured bacterium R35 (AF407690)	*Alkaliphilus*	
	7	193	2.3	Uncultured *Geobacter* OTU6 (FM204962)	*Geobacter*	MFC3
	8	166	2.0	Uncultured bacterium UB106 (AM490695)	*Betaproteobacteria*	
	9	150	1.8	Uncultured bacterium S1-41 (EU015093)	*Dechloromonas*	
	10	128	1.5	Uncultured bacterium LT-SB-B13 (FJ755757)	*Rhodocyclaceae*	
	11	102	1.2	*Rhodocyclus* sp. HOD 5 (AY691423)	*Rhodocyclus*	
	12	95	1.1	Denitrifying bacterium NOB2A10 (FJ802256)	*Rhodocyclaceae*	
	Others	3,947	46.4			
	Total	8,511	100.0			
0.05 M	1	4,051	38.7	*Geobacter* sp. Ply1 (EF527233)	*Geobacter*	MFC2
	2	693	6.6	Uncultured bacterium WCHB1-80 (AF050563)	*Leptolinea*	
	3	614	5.9	Uncultured bacterium Kas165B (EF203202)	*Chlorobiales*	
	4	340	3.2	Uncultured bacterium BS055 (AB240241)	*Geobacter*	MFC4
	5	331	3.2	Uncultured bacterium 613 (FM178812)	*Spirochaetaceae*	
	6	247	2.4	Uncultured bacterium TSAC14 (AB186805)	*Chlorobiales*	
	7	241	2.3	Uncultured bacterium EBL49 (GU591537)	*Sphingobacteriales*	
	8	214	2.0	Uncultured bacterium WCHB1-40 (AF050549)	*Spirochaetaceae*	
	9	133	1.3	*Azospirillum* sp. B510 (AP010946)	*Azospirillum*	
	10	125	1.2	Uncultured bacterium WBB100 (EU184876)	*Rhizobium*	
	11	116	1.1	Uncultured bacterium SJA-87 (AJ009478)	*Holophaga*	
	12	105	1.0	Uncultured bacterium 55c (FJ462089)	*Chlorobiales*	
	Others	3,265	31.2			
	Total	10,475	100.0			
0.1 M	1	8,719	60.1	*Geobacter* sp. Ply1 (EF527233)	*Geobacter*	MFC2
	2	588	4.1	Uncultured bacterium WCHB1-80 (AF050563)	*Leptolinea*	
	3	452	3.1	Uncultured bacterium BS055 (AB240241)	*Geobacter*	MFC4
	4	281	1.9	Uncultured bacterium WBB100 (EU184876)	*Rhizobium*	
	Others	4,460	30.8			
	Total	14,500	100.0			
0.3 M	1	2,433	16.3	*Aeromonas media* NFB-5 (GU810523)	*Aeromonas*	
	2	2,417	16.2	*Aeromonas hydrophila* (X87271)	*Aeromonas*	
	3	1,917	12.8	Uncultured bacterium AKAU4090 (DQ125857)	*Rhodococcus*	
	4	300	2.0	*Ochrobactrum* sp. B2 BBTR46 (DQ337583)	*Ochrobactrum*	
	5	290	1.9	Uncultured bacterium 4A3B3C1 (GU451204)	*Bacteria*	
	6	256	1.7	Uncultured *Bacteroidetes* QEDN10DH05 (CU927327)	*Parabacteroides*	
	7	252	1.7	Uncultured bacterium G3DCM-82 (EU037335)	*Rikenellaceae*	
	8	236	1.6	Uncultured *Bacteroidetes* RsStar205 (AB522124)	*Dysgonomonas*	
	9	234	1.6	Uncultured bacterium SK8EF (AY753402)	*Alkaliphilus*	
	10	228	1.5	Uncultured anaerobic bacterium B-4C (AY953243)	*Dysgonomonas*	
	11	156	1.0	*Azospirillum* sp. YM 274 (GU396258)	*Azospirillum*	
	Others	6,238	41.7			
	Total	14,957	100.0			

NaCl	Order	No. of read	Percent (%)	Closely related sequence (accession no.)	Taxonomy	Description
0.6 M	1	1,146	11.1	*Pseudomonas* sp. × 7 (GQ247888)	*Stenotrophomonas*	
	2	957	9.2	*Pseudomonas putida* (EF526503)	*Pseudomonas*	
	3	940	9.1	*Aeromonas media* NFB-5 (GU810523)	*Aeromonas*	
	4	892	8.6	*Aeromonas hydrophila* (X87271)	*Aeromonas*	
	5	616	5.9	*Pseudomonas oleovorans* RS1 (DQ842018)	*Pseudomonas*	
	6	542	5.2	Uncultured bacterium AKAU4090 (DQ125857)	*Rhodococcus*	
	7	502	4.8	*Pseudomonas* sp. P14 (EF627998)	*Pseudomonas*	
	8	337	3.3	*Ochrobactrum* sp. B2 BBTR46 (DQ337583)	*Ochrobactrum*	
	9	268	2.6	*Pseudomonas fluorescens* LMG 14675 (GU198125)	*Pseudomonas*	
	10	268	2.6	Uncultured bacterium nbw502e10c1 (GQ102022)	*Stenotrophomonas*	
	11	143	1.4	Uncultured bacterium nbt227f06 (EU537939)	*Pseudomonas*	
	12	131	1.3	Uncultured bacterium CHINA11 (GU563744)	*Stenotrophomonas*	
	13	107	1.0	Uncultured *Achromobacter* 13 (FJ195779)	*Achromobacter*	
	Others	3,947	46.4			
	Total	8,511	100.0			
1.8 M	1	5,720	69.2	*Staphylococcus sciuri* (AJ421446)	*Staphylococcus*	
	2	326	3.9	*Dietzia daqingensis* (AY603001)	*Dietzia*	
	3	129	1.6	*Stenotrophomonas* sp. MFC-C (AB183423)	*Stenotrophomonas*	
	4	114	1.4	Uncultured bacterium aab28d03 (DQ819316)	*Staphylococcus*	
	5	92	1.1	Uncultured bacterium AKAU4090 (DQ125857)	*Rhodococcus*	
	Others	1,886	22.8			
	Total	8,267	100.0

## Discussion

The present study shows that interdependencies exist among salt concentration, power outputs, and anode exoelectrogens in MFCs. Clear correlation was detected between the power output and abundance of *Geobacteraceae* bacteria (Figure 5), suggesting that NaCl affects the physiology and growth of these exoelectrogens.

The abundance of *Geobacter* bacteria increased as the NaCl concentration increased from 0 to 0.1 M, while these were markedly decreased above 0.3 M NaCl (Figures 5, 6). This trend is consistent with the fact that members of this family have mostly been isolated from freshwater environments and preferentially grow in freshwater media without NaCl (Lovley et al. 2011). In addition, physiological characterization of *Geobacter* isolates has demonstrated that they tolerate up to 10 g NaCl per liter (0.17 M) (Nevin et al. 2005). Given these features of *Geobacter* bacteria, the low electric outputs at 0.3 M NaCl and higher are likely attributable to the inability of these exoelectrogens to grow at these NaCl concentrations. In contrast, the finding that anode-associated *Geobacteraceae* bacteria preferentially grow at 0.1 M NaCl is notable, and this feature may be specific for those growing by anode respiration in MFCs. This clearly demonstrates that a certain level of the ionic strength (corresponding to the solution conductivity) is required for anode respiration by exoelectrogens. Although a salt-tolerant strain of *Geobacter* that can generate electricity in an MFC at 0.65 M NaCl was recently isolated and characterized (Sun et al. 2014), this strain also preferentially generates electricity at ionic strengths corresponding to 0.1 M NaCl or lower. These observations suggest that the optimum NaCl concentration (0.1 M NaCl in the present study) is determined by the balance of the two factors, namely, the solution conductivity and salt tolerance of exoelectrogens. Future research will examine transcriptomic responses of *Geobacter* exoelectrogens to different concentrations of NaCl.

At NaCl concentrations of 0.3 M and higher, bacteria affiliated with *Gammaproteobacteria* and *Bacilli* were abundantly detected (Figure 6). Major genera in these phyla are *Pseudomonas*, *Aeromomas* and *Staphylococcus*; among these, *Pseudomonas* (Boon et al. 2008) and *Aeromomas* (Pham et al. 2003) are known to include exoelectrogens, while direct electricity generation by species of *Staphylococcus* has not been reported. Although electric outputs at these NaCl concentrations were low, it may be interesting to isolate these bacteria for examining their abilities to generate electricity in MFCs at high salt concentrations.
